# Computing Shor’s algorithmic steps with interference patterns of classical light

**DOI:** 10.1038/s41598-022-25796-w

**Published:** 2022-12-07

**Authors:** Wei Wang, Ziyang You, Shuangpeng Wang, Zikang Tang, Hou Ian

**Affiliations:** Institute of Applied Physics and Materials Engineering, University of Macau, Macau S.A.R, China

**Keywords:** Quantum physics, Quantum information, Quantum optics

## Abstract

When considered as orthogonal bases in distinct vector spaces, the unit vectors of polarization directions and the Laguerre–Gaussian modes of polarization amplitude are inseparable, constituting a so-called classical entangled light beam. Equating this classical entanglement to quantum entanglement necessary for computing purpose, we show that the parallelism featured in Shor’s factoring algorithm is equivalent to the concurrent light-path propagation of an entangled beam or pulse train. A gedanken experiment is proposed for executing the key algorithmic steps of modular exponentiation and Fourier transform on a target integer *N* using only classical manipulations on the amplitudes and polarization directions. The multiplicative order associated with the sought-after integer factors is identified through a four-hole diffraction interference from sources obtained from the entangled beam profile. The unique mapping from the fringe patterns to the computed order is demonstrated through simulations for the case $N=15$.

## Introduction

Non-zero correlation between space-like events was initially debated in the famous Einstein–Podolsky–Rosen thought experiment^1^. This contemplated correlation was later quantified by Bell’s inequality^2^ and CHSH inequality^3^, which subsequently give rise to the concept of quantum entanglement^4^. Violating the law of locality, entanglement has long been thought of as a unique feature only for systems operating in the quantum regime^5–7^. But recent investigations have revealed that entanglement to some partial extent can be emulated in classical systems^8–10^. In particular, the classical entanglement between the polarization direction and the polarization amplitude is experimentally verified^11^; so is that between the polarization and the Orbital Angular Momentum (OAM) in a Laguerre–Gaussian (LG) beam^12^.

Entanglement plays a key role in implementing quantum algorithms. The celebrated Shor’s algorithm relies on the entanglement of two quantum states, guised as two computational registers, to speed up the finding of the correct factor of a large integer^13^. The two registers can be physically realized by superconducting qubits^14^, nuclear spins^15^, or optical photons^16–20^. Compared to quantum entanglement exemplified in these quantum systems, classical entanglement obtains a similar correlation level while having additional merits in coherence, generation, and detection^21,22^, making classical entangled systems substitute the roles of quantum entangled states in communication and computation^23–25^. In this work, we apply the concept of classical entanglement to propose a gedanken experiment using entirely classical sources and optical elements to study the realizability of Shor’s algorithm.

The existence of classical implementations of Deutsch algorithm^26^, quantum walk^27^, and quantum Fourier transform (QFT)^12^ shows that quantum algorithms do not necessarily require a quantum system to be realized. Rather, the border line that divides quantum mechanics from classical mechanics does not exactly coincide with the boundary between quantum and classical algorithms. The fully quantum optical versions of Shor’s algorithms^16^ generate entangled pairs of photons, which are let undergo probabilistically selective paths with computational significance before one photon is projectively measured and its entangled counterpart collapses into the desired state. Here, we make use of the intrinsically entangled degrees of freedom in the OAM amplitude modes and the polarization directions of an LG beam to demonstrate an equivalent implementation of the computational steps used by Shor’s algorithm. The beam carries out the parallel computational process by running a set of split beams concurrently through multiple viable light paths before they are congregated to generate a unique interference pattern^28^ mappable to the computation result. From this classical perspective, an equivalence between the algorithmic complexity and the optical-path complexity is established.

In particular, the OAM modes are used to represent the computational basis of the control register while the polarization directions those of the work register. The latter would store the data computed from modular exponentiation and discrete Fourier transform, the two key steps of Shor’s algorithm. After the two-step process, a four-hole interference setup sources from the unified beam profile a distinguishable fringe pattern that has a unique correspondence to a specific entangled computational bases in the two registers. We demonstrate this distinguishabililty below of distinct multiplticative orders from fringe patterns for the illustrative case of $N=15$ where only 4 OAM modes are needed. To scale up the applicability for a larger *N*, we show that the same setup can be used with a continuous-wave laser source replaced by a pulse train. In the last part, we prove that the complexity of our method is consistent with the original Shor’s algorithm.

In the following, we present the algorithmic steps based on LG beams in “Classical entanglement and shor’s algorithm” section. The optical path designs are explained in “Optical paths for algorithmic steps” section and the detection methods discussed in “Detection method and results” section. In “Complexity analysis” section analyzes the complexity. Conclusions are given in “Conclusions” section.

## Classical entanglement and Shor’s algorithm

Classical entanglement refers to non-separable correlations among the degrees of freedom in classical optics. For instance, the two orthogonal polarization directions (${\textbf{e}}_{\textbf{x}}$ and ${\textbf{e}}_{\textbf{y}}$) and their respective field amplitudes ($E_{x}$ and $E_{y}$) can be taken as an entangled pair in the polarized optical field ${\textbf{E}}=E_{x}{\textbf{e}}_{\textbf{x}}+E_{y}{\textbf{e}}_{\textbf{y}}$. Allowing the field amplitudes to vary spatially beyond those depicted by plane waves, one arrives at linearly polarized LG beams

1 $$\begin{aligned} {\textbf{E}}=u_{p,l}{\textbf{H}}+u_{p,-l}{\textbf{V}}, \end{aligned}$$

where ${\textbf{H}}$ and ${\textbf{V}}$ denote the unit vectors for respectively horizontal and vertical polarization directions while

2 $$\begin{aligned} u_{p,l}\left( r,\varphi ,z\right)&=\frac{C\left(r\sqrt{2}/w_{z}\right)^{\left| l\right| }}{\sqrt{1+z^{2}/z_{R}^{2}}}e^{-r^{2}/w_{z}^{2}}L_{p}^{\left| l\right| }\left( \frac{2r^{2}}{w_{z}^{2}}\right) \nonumber \\&\quad \times e^{-il\varphi }\exp i\left\{ \frac{kr^{2}z}{2\left( z^{2}+z_{R}^{2}\right) }+\left( 2p+\left| l\right| +1\right) \tan ^{-1}\frac{z}{z_{R}}\right\} \end{aligned}$$

indicate the distribution function for the OAM-mode amplitudes in cylindrical coordinates^29^ with *z* being the propagation direction. In the equation, *C* is a normalized constant, $z_{R}$ the Rayleigh range, $w_{z}$ the beam waist, and $L_{p}^{\left| l\right| }$ the associated Laguerre polynomial. It is worth noting that the function has an azimuthal angular dependence of $\exp (-il\varphi )$, where *l* is the topological charge determining the angular momentum ($l\hbar$ per photon) and the chirality of helical phase fronts (sign of *l*).

The set of OAM-modes specified by the integer *l* forms an orthogonal set of basis functions for a complete Hilbert space, i.e. the inner product $(u_{p,l},u_{p,l'})=\delta _{l,l'}$. Hence, this OAM space is mathematically isomorphic to a finite dimensional quantum state space and we can use Dirac bra–ket notation for state $\left| l\right\rangle$ to indicate the OAM mode $u_{p,l}$. Similarly, the two polarization directions are orthogonal, which forms a two-dimensional space independent from the OAM modes and prompts us to use $\left| H\right\rangle$ and $\left| V\right\rangle$ to denote ${\textbf{H}}$ and ${\textbf{V}}$. We make use of these orthogonalities to encode the computational bases of two registers, a control register $\left| \psi _{C}\right\rangle$ and a work register $\left| \psi _{W}\right\rangle$, that Shor’s algorithm calls for.

After initialization, the joint computer state is separable, i.e.$\left| \psi _{C}\right\rangle \otimes \left| \psi _{W}\right\rangle$. The first algorithmic step^30^ is the formation of the entangled state

3 $$\begin{aligned} \frac{1}{\sqrt{2^{n}}}\sum _{x=0}^{2^{n}-1}\mathop {\left| x\right\rangle \left| a^{x}{\text {mod}}N\right\rangle } \end{aligned}$$

amongst the two registers, where the work register encodes the results of modular exponentiation function (MEF) according to the exponent *x* given in the control register and the modulus *N*. The integer *N* is the target integer to be factored and the base $a<N$ is a pre-selected number coprime with *N*, i.e. $\gcd (a,N)=1$. Number-theoretically speaking, factorizing *N* corresponds to finding an *x* value such that it becomes the multiplicative order of the MEF. The candidate of this order *x* lies within the range $\{0,1,\dots ,2^{n}-1\}$, hence the range of the summation, where *n* denotes the number of qubits necessary for constructing each register and is determined by *N*, i.e. $2^{n}\approx \sqrt{N}$. Taking $N=15$ as an example, one would have $n=2$ and $a=11$, making $x\in \{0,1,2,3\}$ while the exponentiation assumes two possible values, 1 and 11.

**Figure 1:**
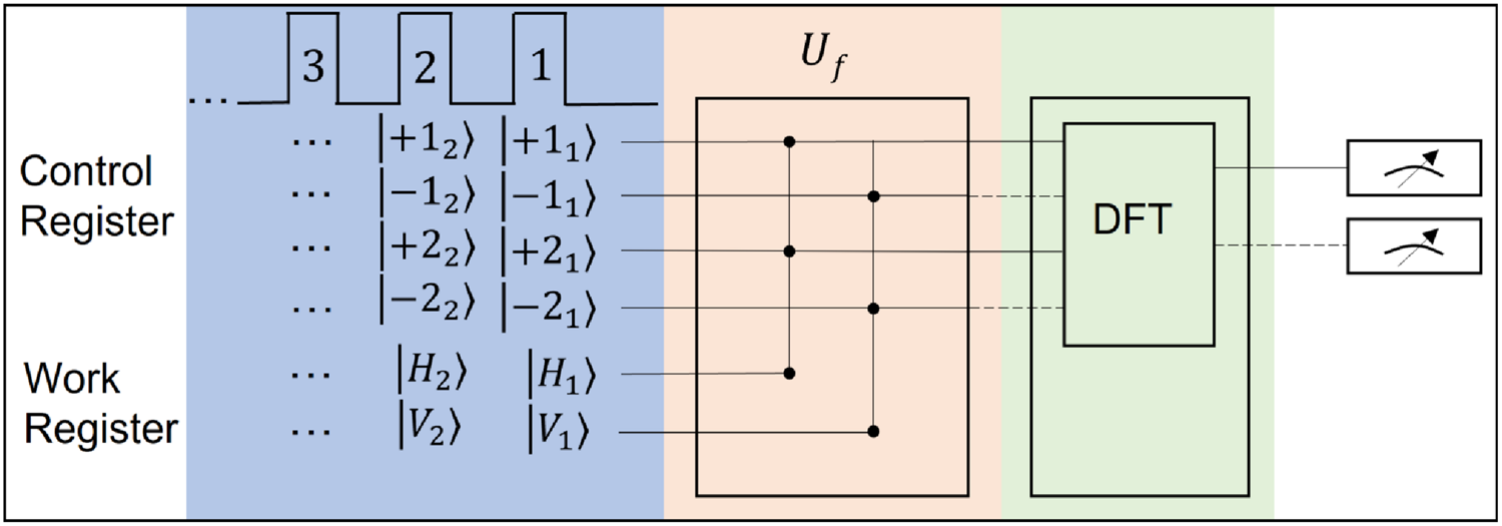
Circuit flow diagram for implementing Shor’s algorithm. The blue, pink, and green parts indicate the stages for initialization, modular exponentiation, and discrete Fourier transform, respectively. The blue arrow in MEF in this part refers to the polarization flip. The modular exponentiation is implemented across the control and the work registers by a unitary transform $U_{f}$, before which one eigenstate of the work register is flipped (shown as the blue arrow) and after which the eigenstates across the two registers are distinctly entangled (shown by the black nodes). The two entangled states associated with $\left| H\right\rangle$ and $\left| V\right\rangle$ directions in each pulse are separately passed on to the Fourier transform stage, respectively indicated by the solid and the dashed lines.

To establish the state of Eq. (3) in a classical beam of light, we store the control register information $\psi _{C}$ into the OAM-modes of an LG bream and work register information $\psi _{W}$ into the polarization directions ${\textbf{H}}$ and ${\textbf{V}}$ of that beam. Hence, the initial separable input state can be written as

4 $$\begin{aligned} \left| \psi _{\textrm{in}}\right\rangle =\sum _{l=1}^{k}\left[ \left| +l\right\rangle +\left| -l\right\rangle \right] \otimes \left| H\right\rangle , \end{aligned}$$

where the normalization constant is omitted to simplify the expression. For an *n*-bit long integer *N*, *k* should at least be equal to $2^{n-1}$. Then, all algorithmic steps involved for computing the multiplicative order described by the originally quantum algorithm can be realized on Eq. (4) using just classical light path manipulations. Figure 1 shows the partitioning of the four major steps of Shor’s algorithm: (1) preparing the initial state; (2) performing modular exponentiation (MEF); (3) performing quantum or discrete Fourier transform (DFT); and (4) executing read out.

Before describing the detailed setup of an integrated light path for carrying out the forementioned four steps, we examine from a formal perspective how Eq. (4) changes in each stage to demonstrate that the final state of the LG beam contains computationally significant information. First, one notes the work register is limited to two states $\left| H\right\rangle$ and $\left| V\right\rangle$. To resolve this scaling limitation, we appeal to a pulse train implementation where the number of pulses required to encode the modulus of the control register is, in the worst case scenario where *N* is prime, *n*. On average, if a factor exists for *N*, only $n-1$ bits or pulses are needed. In the meanwhile, the extention in pulse number allows the control register information be distributed among multiple OAM modes across pulses. Therefore, in general, one encodes the state

5 $$\begin{aligned} \left| \psi \right\rangle =\sum _{l=1}^{k}\prod _{i=1}^{\otimes n}\left[ \left| +l_{i},H_{i}\right\rangle +\left| -l_{i},V_{i}\right\rangle \right] , \end{aligned}$$

in an *n*-pulse train.

**Figure 2:**
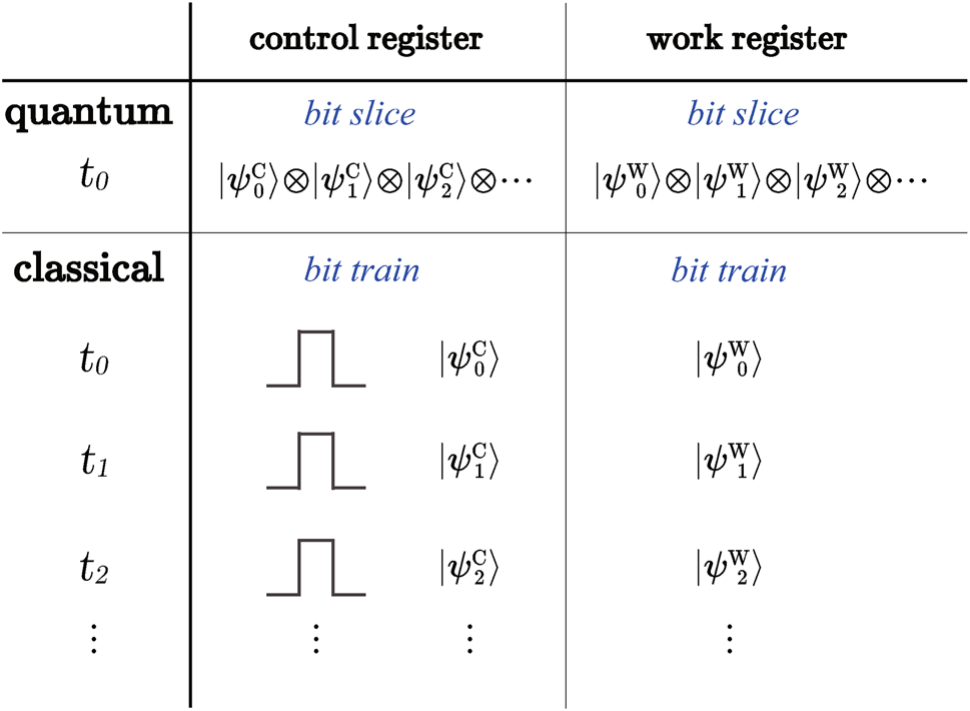
Illustration for the distinct data encoding schemes used by two entangled registers in typical quantum entangling implementations and our classical entangling implementation. In the former, the qubits of the control register are collectively entangled with the qubits of the work register; the bit-slice data in the two registers are entangled as cluster entangled states. In the latter, since the pulses are time ordered without inter-pulse interactions, the entangling encoding extends in time and is done one bit in the control register with one bit in the work register at a time. The time ordering is equivalent to spatial ordering in a light space and does not contribute to extra time complexity of the algorithm.

Note that the encoding scheme of Eq. (5) is different from typical encoding schemes used for implementing Shor’s algorithms. As illustrated in Fig. 2, the bits in both the control and the work registers are temporally ordered to comply with the time-ordered optical pulses in a pulse train. For the simplest case where two OAM modes are used to entangled with the polarization directions, one pulse encodes the entangled data of exactly one bit in $\left| \psi _{C}\right\rangle$ and one bit in $\left| \psi _{W}\right\rangle$. The pulse train extends in time to encode the multi-bit data necessary for both registers. In contrast, conventional approaches on solid-state qubits and photon pairs have the scaling done across space to encode entangled bit slices collectively. One bit-slice data is encoded as a cluster state in $\left| \psi _{C}\right\rangle$ to entangle with a cluster state in $\left| \psi _{W}\right\rangle$ containing the data of an associated bit slice. This distinction between vertical extension and horizontal scaling affects the detection scheme at eventual readout. The quantum projection or collapse measurement is conducted, theoretically, in one shot, whereas the pulse train scheme requires bit-by-bit detection on successive pulses. However, pulse detection on photo diodes occupies fixed time. Algorithmically speaking, the complete decoding of a pulse train is completed within one iteration; the extra complexity incurred is the constant ${\mathcal {O}}(1)$. Therefore, the new encoding scheme does not sacrifice extra algorithmic complexity to the factoring procedure.

The modular exponentiation $a^{x}\mod N$ establishes a specific correlation between the computational bases in the control and work registers. For example, if the OAM modes are limited to $\left\{ +1,-1\right\}$, the register encoding becomes fully binary and one should have

6 $$\begin{aligned} \left| \psi _{\textrm{MEF}}\right\rangle =\sum _{j=1}^{n}\left| x_{j},a^{x_{j}}\mod N\right\rangle \end{aligned}$$

after the exponentiation step, where $x_{j}$ indicates a binary sequence encoded by the two OAM modes.

For the case $N=15$, the MEF should set work register to 1 ($\left| \psi _{W}\right\rangle =\left| H\right\rangle$) that associates with either the case $x=0$ or $x=2$, i.e.$\left| \psi _{C}\right\rangle =\left| +1\right\rangle$ or $\left| +2\right\rangle$, at the control register; complementarily, the work register stores 11 ($\left| \psi _{W}\right\rangle =\left| V\right\rangle$) for $x=1$ or $x=3$ at the control register, i.e. $\left| \psi _{C}\right\rangle =\left| -1\right\rangle$ or $\left| -2\right\rangle$. The entangled state after the MEF should read

7 $$\begin{aligned} \sum _{l=1}^{2}\left[ \left| +l,H\right\rangle +\left| -l,V\right\rangle \right] , \end{aligned}$$

where the necessary information for eliciting the correct multiplicative order is contained. Therefore, using four OAM modes, one pulse from the source and a CW laser is sufficient to encode for $N=15$.

The next step in Shor’s original routine involves a projective readout on Eq. (7) that collapses the work register into a definite $\left| H\right\rangle$ or $\left| V\right\rangle$ state, leaving the control register retain an eigenstate superposition. Note that the MEF entanglement guarantees that either way of the collapse will impose the same multiplicative order in the superposition. In contrast, deprived of the unique property of quantum collapse, classical beams can substitute this step by simply splitting the mixed beam through a polarizing beam splitter (PBS). That is, the PBS would split the pulse sequence of Eq. (6) into two, each having the same polarization direction among every pulse, e.g. one would have $\left| \psi _{\textrm{COL}}\right\rangle =\sum _{r}\left| x_{r},{\mathcal {P}}\right\rangle$ where $\left| {\mathcal {P}}\right\rangle$ is either $\left| H\right\rangle$ or $\left| V\right\rangle$ and $x_{r}=qr+m$ (*m* is the modulus and *q* is the quotient).

In the $N=15$ example, collapsing $\left| \psi _{W}\right\rangle$ into either $\left| H\right\rangle$ or $\left| V\right\rangle$ will retain identically two eigenstates in $\left| \psi _{C}\right\rangle$; whether either $\left| +1\right\rangle +\left| +2\right\rangle$ or $\left| -1\right\rangle +\left| -2\right\rangle$ points to the same order 2 (i.e. $11^{2}\mod 15=1$).

8 $$\begin{aligned} \left| \psi _{H}\right\rangle&=\left| +1,H\right\rangle +\left| +2,H\right\rangle ,\nonumber \\ \left| \psi _{V}\right\rangle&=\left| -1,V\right\rangle +\left| -2,V\right\rangle , \end{aligned}$$

on either of which the subsequent algorithmic steps can operate to achieve integer factoring of *N*.

One of the polarized beam in Eq. (8) then undergoes the discrete Fourier transform (DFT) (the third major key illustrated in Fig. 1) expressed as

9 $$\begin{aligned} \left| x\right\rangle \rightarrow \mathop {\frac{1}{\sqrt{2^{n}}}\sum _{j=0}^{2^{n}-1}}e^{i2\pi jx/2^{n}}\left| j\right\rangle . \end{aligned}$$

Each state $\left| x\right\rangle$ is transformed to a set of new orthogonal bases $\left| j\right\rangle (j\in \{0,1,\dots ,2^{n}-1\})$ with a specific distribution of phases determined by the value of *x* in $2^{n}$ dimensions. For typical quantum optical^19,20^ or superconducting circuit^14^ implementations, Hadamard gates or control phase gates are consecutively applied to individual qubits to realize the Fourier transform, making the transform of Eq. (9) essentially an *n*-step one-qubit operation. The OAM space of the LG beam considered here need not be decomposed into 2-level spaces, so the phases can be imposed concurrently on the OAM modes $\left| l\right\rangle$, i.e. the DFT transform bases $\left| j\right\rangle$, once they are separated. In the illustrated example, since $\left| j\right\rangle$ ranges over $\left| +1\right\rangle$, $\left| -1\right\rangle$, $\left| +2\right\rangle$ and $\left| -2\right\rangle$, the intermediate state resulted from $\left| l=-1\right\rangle$ for example becomes

10 $$\begin{aligned} \left| \psi _{\textrm{int}}^{(-1)}\right\rangle =\left| +1\right\rangle +i\left| -1\right\rangle -\left| +2\right\rangle -i\left| -2\right\rangle . \end{aligned}$$

The states with phases $\{0,\pi /2,\pi ,3\pi /2\}$ for different *j* imposed can be realized by spiral phase plates (SPP) and phase-only spatial light modulators (SLM) as well as q-plates^31^ and digital micromirror devices (DMD)^32^. While the former performs the function of converting OAM modes, e.g. from $\left| +1\right\rangle$ to $\left| +2\right\rangle$^33^, the latter introduces an arbitrary phase to a specific mode. Combining the two allows one to generate $i\left| +1\right\rangle$ from $\left| -1\right\rangle$ through the addition of a $\pi /2$ phase^12^. To complete the DFT transform, each state that underwent Eq. (9) should be combined through addition, i.e. to arrive at

11 $$\begin{aligned} \left| \psi _{\textrm{DFT}}\right\rangle =\left| \psi _{\textrm{int}}^{(\pm 1)}\right\rangle +\left| \psi _{\textrm{int}}^{(\pm 2)}\right\rangle , \end{aligned}$$

where the sign depends on which beams we choose from Eq. (8).

For the demonstration case $N=15$, where the choice of the sign would lead the control register to retain either the superposition $\left| +1\right\rangle +\left| +2\right\rangle$ or $\left| +1\right\rangle -\left| +2\right\rangle$. The phase difference between the two basis vectors does not affect the computation significance. Either state contains the OAM modes $l=+1$ and $l=+2$, which translate into the computational values 0 and 2, respectively, according to the algorithmic premise we have assumed. Since only a non-zero value is acceptable, one is left with $x=2$ from which the multiplicative order $r=2^{n}/x$ can be obtained. Then, the integer factors become apparent by finding the greatest common divisor of $11^{r/2}\pm 1$ and *N*. The last two conversion steps are not computationally complex and thus not necessarily executed in the light paths.

## Optical paths for algorithmic steps

The two major algorithmic steps according to Fig. 1 and the discussion in the last section are: (1) modular exponentiation function (MEF) and (2) discrete Fourier transform (DFT). Below we examine the details of the experimental setup that can accomplish these two steps.

### Modular exponentiation

**Figure 3:**
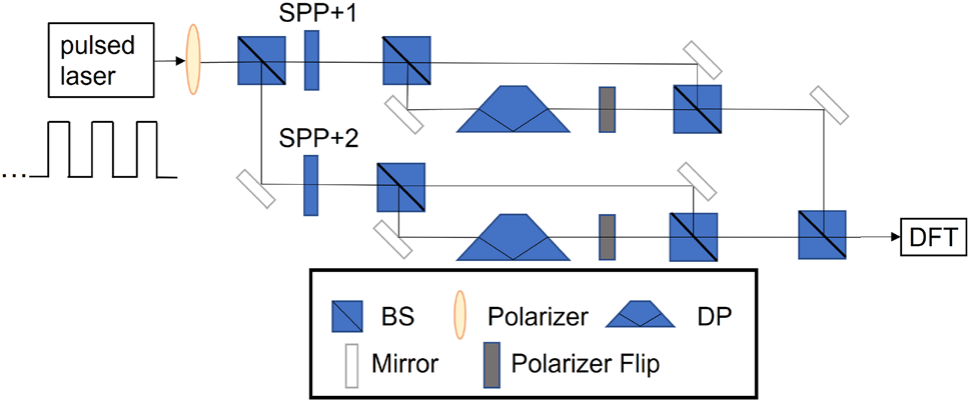
Optical path diagram of modular exponentiation in Shor’s algorithm. A laser pulse train passes through a polarizer and SPPs to generate the OAM modes of Laguerre–Gaussian beams, which refer to different states in the algorithm. The DPs, polarizer flips, and BSs are used to execute the algorithm. BS indicates 50/50 beam splitters, SPP spiral phase plates, and DP Dove prisms.

The optical path setup for modular exponentiation is shown in Fig. 3. The light source is a phase-locked laser pulse train, which is let pass through a horizontal polarizer and split into two equal intensity branches of the same horizontal polarization along two paths. These two paths are identical except for the spiral phase plates (SPP) which modulate identical beams into distinct OAM modes. For the exemplifying $N=15$ case, one could consider a train of one single pulse or, more simply, a CW laser as the source and have OAM modes of $l=+1$ and $l=+2$^33^ representing the computational states $\left| +1,H\right\rangle$ and $\left| +2,H\right\rangle$, respectively. The beam along each path then passes through a beam splitter (BS), from which one branch goes through a Dove prism (DP) to invert the OAM sign ($+1\rightarrow -1$ or $+2\rightarrow -2$)^34^ and a polarizer set to flip $\left| H\right\rangle$ to $\left| V\right\rangle$, while the other branch remains unchanged. Eventually, the four branches of the two beams are recombined using three beam splitters, giving rise to the desired state after MEF according to Eq. (7).

### Discrete Fourier transform

As explained in the last section, the classical light path needs to achieve an effect analogous to quantum collapse to elicit pulse states useful for computational purposes before feeding them into subsequent stages for discrete Fourier transforms. To do this, the output from the MEF stage is split into two through a polarizer, allowing one to obtain two pulse trains, each carrying the same polarization direction for all pulses whose OAM modes are either $\{\left| +1\right\rangle ,\left| +2\right\rangle ,\dots \}$ or $\{\left| -1\right\rangle ,\left| -2\right\rangle ,\dots \}$. Each train is further split by an OAM sorter^35^ to obtain individual pulses of particular $\left| \pm j\right\rangle$. The DFT process is effectively a transformation that imposes specific phases on each pulse. Realized by phase gates on photonic systems, it is in contrast realizable using Sagnac interferometry here for the classical light beams. Each pulse corresponding to a particular $\left| j\right\rangle$ is first superposed with its inverse state $\left| -j\right\rangle$ through beam splitters and a Dove prism before it enters its designated interferometer, in which $\left| j\right\rangle$ generates a superposition of all basis states of the same sign.

**Figure 4:**
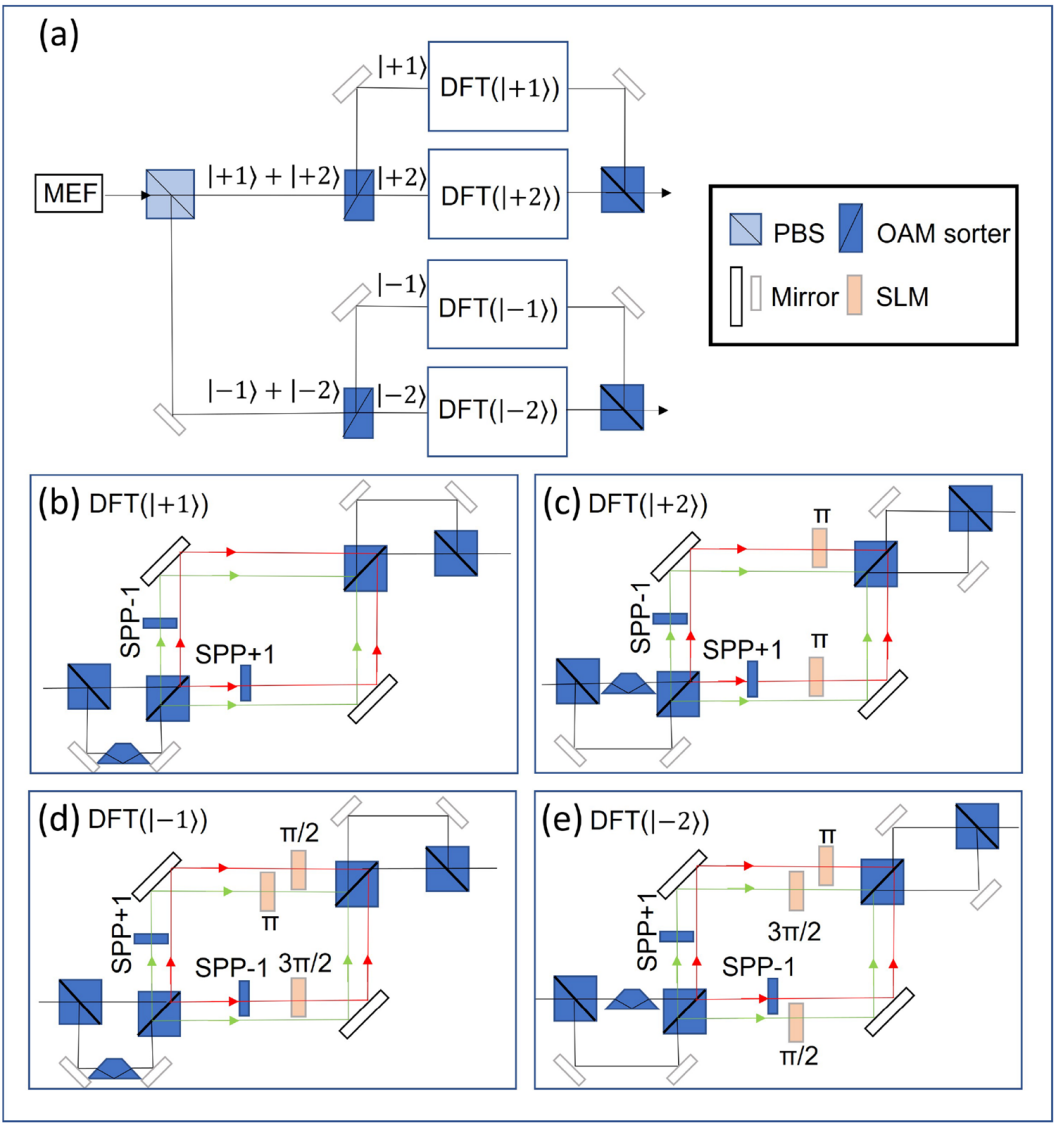
Optical path diagram of DFT in Shor’s algorithm. The work register is measured to different polarization directions by a PBS. Each beam is sorted by an OAM sorter into two beams with different OAM and then emulates the DFT individually. Finally combining the beams and getting the output related to the order. SLM is phase-only spatial light modulator.

For a concrete illustration, consider the optical path setup shown in Fig. 4 for $N=15$ with the input MEF end being the states given in Eq. (8). The beam is first polarizingly split before fed into OAM sorters, producing in total four branches of distinct states, i.e. $\left| +1\right\rangle$, $\left| -1\right\rangle$, $\left| +2\right\rangle$, and $\left| -2\right\rangle$. Considering all these states running in parallel, DFT effectively implements a $4\times 4$ matrix transformation on them, where the matrix entries represent complex phase coefficients of a Fourier transform. To realize the resulting state in Eq. (11), the Sagnac interferometers, shown as boxes of Fig. 4, use SPPs to generate new OAM basis, e.g. $\left| +1\right\rangle \rightarrow \left| +1\right\rangle +\left| +2\right\rangle$ and $\left| -1\right\rangle \rightarrow \left| -1\right\rangle +\left| -2\right\rangle$, whilst using the SLMs subsequently to generate corresponding phases along the state-designated paths. The beams are eventually combined using BS to output the computed state as in Eq. (11). This state then enters the final stage of intereference fringe detection to obtain the information of multiplicative order.

## Detection method and results

**Figure 5:**
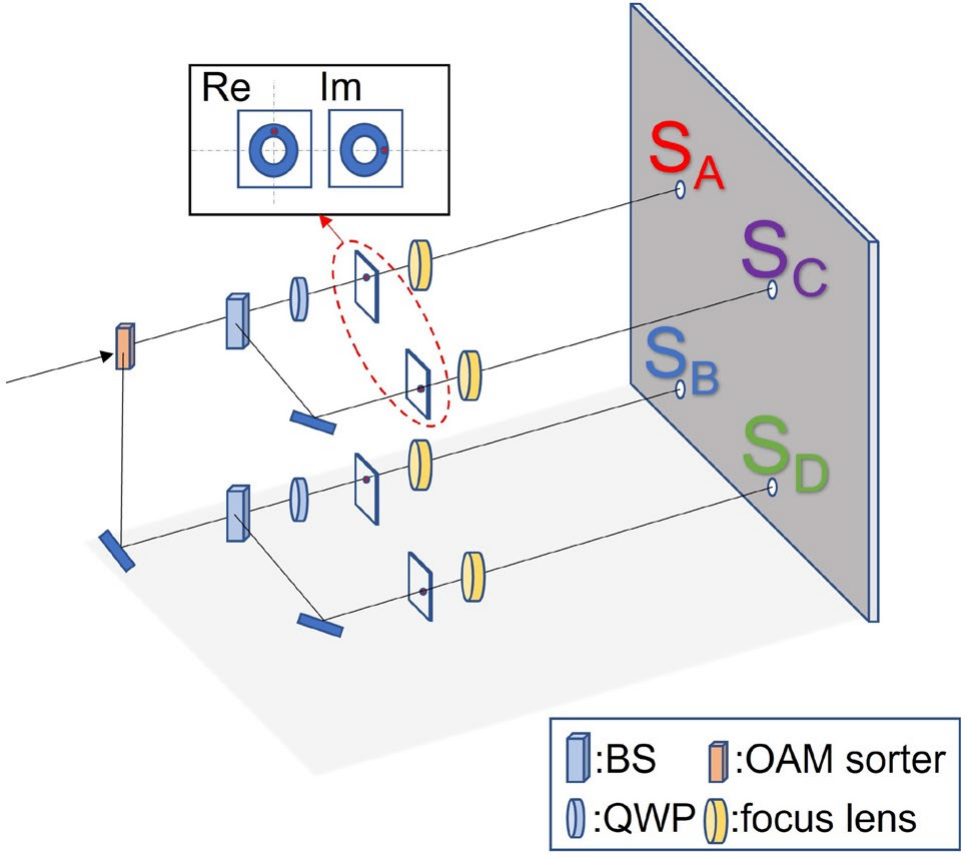
Optical path diagram of the detection method. The DFT output beam first goes through a OAM sorter to separate the beam into different LG modes. Then two beams are decomposed into four beams by two BSs. By the orthogonal aperture diaphragms, the LG beams are separated into the real and imaginary components. Finally, refocused beams enter the four holes as the interference sources. The elements in the upper box represent the orthogonal aperture diaphragms, BS is beam splitter, QWP is quarter-wave plate.

The DFT stage outputs a train of pulses containing different OAM states with computational values. The last step of the setup is light paths that are responsible for telling what computational states are contained in these LG beams. To visually discern the computational states, we follow a proposal given in Ref.^28^ to let the distinct OAM states interfere with each other, generating unique fringe patterns that are one-one corresponding to the quantum states. In other words, the desired multiplicative order is implied in a two-dimensional fringe image projected on a screen, as illustrated in Fig. 5. Having a pulse train to generate the interference implies the fringe patterns be recorded over multiple frames, where the frame rate corresponds to the pulse width and the pulse period. Since for $N=15$, one frame is sufficient, we illustrate the interference generation below with a CW laser source in mind.

To implement the interference apparatus, the output from DFT is fed into an OAM sorter to obtain two beams with different *l* modes. Then along each beam path, we use a BS to further split it into two equal parts, constituting four beam paths. Two quarter-wave plates (QWP) are used to compensate for the phase shift introduced by BSs. One orthogonal aperture diaphragm is placed at each transmitted path to select two points from the real and imaginary components of each OAM state. For example, for the LG beam in Eq. (2), the intensity distribution is a helical doughnut with a phase dependence of exp$(-il\varphi )$. The choice of real and imaginary parts depends on the azimuth $\varphi$, which is shown in the illustration of Fig. 5. Two points at the vertical and horizontal axis of the intensity distribution of two equal beams are chosen as the real and imaginary parts, respectively. Finally, the deflected beams from the holes are refocused, acting as the four sources $S_{A}$, $S_{B}$, $S_{C}$ and $S_{D}$ for the four-hole interference. For better interference effect, motorized translation stages (not drawn in the figure) can be used to synchronize the delays among paths.

**Figure 6:**
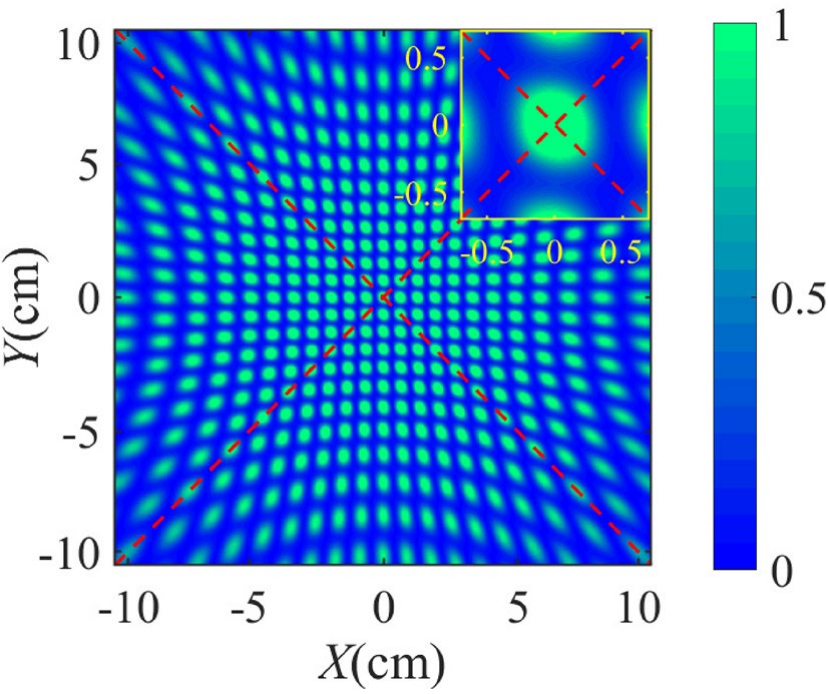
Interference patterns after DFT for state $\left| +1\right\rangle$ $+$ $\left| +2\right\rangle$ in the case $N=15$. $\left| +1\right\rangle$ and $\left| +2\right\rangle$ means 0 and 2 respectively. 0 is obviously invalid. It means order $r=2$.

**Figure 7:**
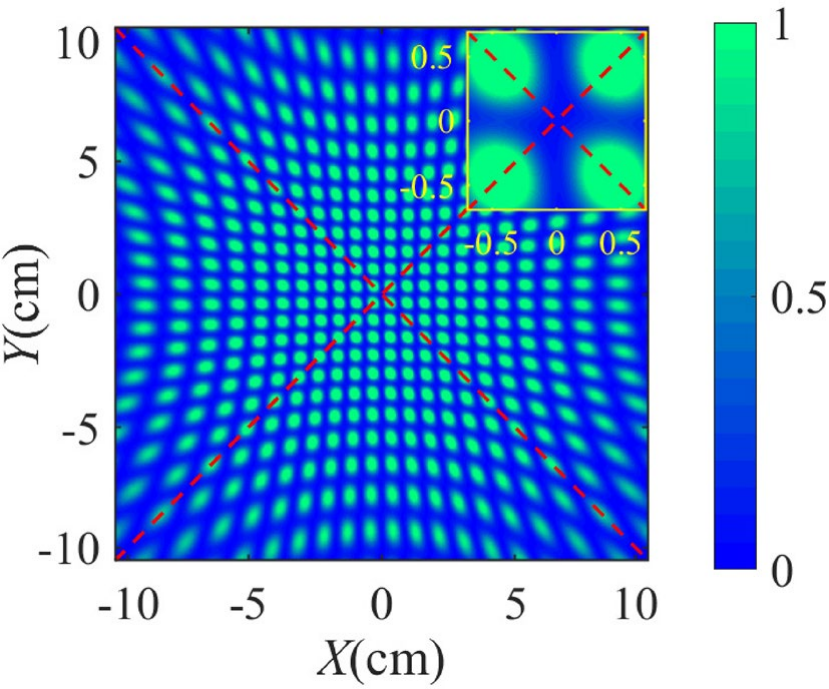
Interference patterns after DFT for state $\left| +1\right\rangle$ $-$ $\left| +2\right\rangle$ in the case $N=15$. It also means order $r=2$.

We simulate the interference patterns by encoding the four light sources. A source laser wavelength of 632 nm, a hole to hole distance of $10\,\upmu$m, a hole to screen distance of 10 cm, and a screen area of $10\times 10\,{\textrm{cm}}^{2}$ are assumed. Meanwhile, we set the $z=0,p=0$ in Eq. (2) for the source beam for simplification. The interference fringe patterns are shown in Figs. 6 and 7.

The pattern in Fig. 6 indicates the first result for the state of Eq. (11) after DFT in upper path of Fig. 4a, which is $\left| +1\right\rangle$ $+$ $\left| +2\right\rangle$. We can see that the pattern is centrosymmetric, where the central area is a bright spot. With these special properties, we can identify the corresponding states in specific experiments. According to the definition of DFT bases in Eq. (9), $\left| +1\right\rangle$ and $\left| +2\right\rangle$ refer to 0 and 2, respectively. The order cannot be 0, so if the pattern in Fig. 6 appears in the experiment, we can extract effective information, that is, order $r=2$.

Figure 7 shows another case in lower path of Fig. 4a after DFT. It refers to the result $\left| +1\right\rangle -\left| +2\right\rangle$. The resulting fringe is still centrosymmetric but with a dark area in the center. This pattern also corresponds to order $r=2$. As opposed to quantum detection, our method can detect the different states in Eq. (11) simultaneously only if we connect a detection system behind the multiple output optical paths in Fig. 4. This is because that the probabilistic solution in the quantum implementation can be equivalent to the probability of choosing one or more of the outputs. Therefore, we can determine the reliability of the results by detecting multiple outputs.

## Complexity analysis

We compare the complexity of the best classical number field sieve algorithm and the original Shor’s algorithm for factoring *N* with our method in the Table 1. Firstly, It has been proven that both the memory space and time of number field sieve is exponential with *N*^36,37^. This means that the computing resources and time required will increase significantly as *N* increases. While quantum bits with supersition and entanglement properities are used to decompose large numbers in polynomial time in original Shor’s algorithm. Basically, It needs $3\lceil {\log }_{2}N\rceil$ qubits in two registers and $O(({\log }N){}^{2}({\log \log }N)({\log \log \log }N))$ steps^30,38^.

**Table 1:** Complexity comparison of number field sieve, Shor’s algorithm and our method for factoring *N*.

	Memory space	Time complexity	References
Number field sieve	${\exp }\left\{ \frac{c}{2}({\log }N)^{1/3}({\log }{\log }N)^{2/3}\right\}$ bits	$O({\exp }\left\{ c({\log }N)^{1/3}({\log }{\log }N)^{2/3}\right\} )$	^36,37^
Original Shor’s algorithm	$3\lceil {\log }_{2}N\rceil$ qubits	$O(({\log }N){}^{2}({\log }{\log }N)({\log }{\log }{\log }N))$	^30,38^
Our method	$\lceil {\log }_{2}N\rceil$ pulses	$O(({\log }N){}^{2}({\log }{\log }N)({log}{\log }{\log }N))$	this work

In contrast, our method only needs $\lceil {\log }_{2}N\rceil$ pulses in Eq. (5) with the advantange of OAM mode and time degree of freedom. In each pulse, we can use the OAM modes and polarization directions to encode two registers respectively. Also, these two degrees of freedom in different pulses can form a binary sequence in a extensible version. For the equivalent time complexity in our method, the MEF adopts the direct encoding for the function result obtained from the external computing platform such as a field-programmable gate array (FPGA). So the time complexity of MEF is similar to the original Shor’s algorithm. Considering the time complexity of each optical element as *O*(1), the DFT we used in the algorithmic step has the time complexity $O({\log }N)$ with the advantage of parallel computing in Fig. 4. Therefore the total time complexity is still consistent with the original Shor’s algorithm. In brief, our macroscopic algorithmic steps using classical light achieve the same complexity as Shor’s algorithm on the basis of ensuring space consistency in order of magnitude.

## Conclusions

In summary, we have implemented the major computational steps in Shor’s algorithm using a classical optical system. The classical entangled pair of OAM mode and polarization in LG beams or pulse trains are used as the control and the work registers, respectively. A detection method using four-hole interference for the entangled states is demonstrated to obtain the multiplicative order *r*, which is equivalent to having found a factor once Euclid’s classical gcd algorithm is applied. Sourcing the interfering light beams from fixed locations of the beam profiles, we have shown that the multiplicative order is derivable from the unique fringe patterns.

Compared to its quantum counterpart, the classical approach avoids the implementations of time-based quantum gates while maintain the same time complexity. The conditions for the generation, operation, and detection of milliwatt-level light pulses are much less stringent, compared to the implementations using single photon pairs, which have low generation and detection efficiency. The former is therefore less expensive to implement and more scalable. In addition, the coherent laser beams is less prompt to the decoherence and noisy environments suffered by superconducting circuits. Nevertheless, since the data are encoded in the time-ordered pulses, the detection through the interference scheme requires the effort to decode the information from complex 2-dimensional images. Hence, future works would be devoted to the simplification of the detection methodology.

From an information and computational perspective, the work here extends the boundary of classical optics into the quantum regime. Like the other works on emulating quantum behavior with classical optics systems, it shows the line separating the quantum from the classical world is less defined as physicists usually thought.

## Data Availability

The datasets used and/or analyzed during the current study are available from the corresponding author on reasonable request.
